# Insecticide susceptibility status of *Anopheles gambiae* (*s.l.*) in South-West Cameroon four years after long-lasting insecticidal net mass distribution

**DOI:** 10.1186/s13071-018-2979-1

**Published:** 2018-07-04

**Authors:** Stravensky Térence Boussougou-Sambe, Wolfgang Ekoko Eyisap, Geraud Canis Taboue Tasse, Stanislas Elysee Mandeng, Lili Ranaise Mbakop, Peter Enyong, Josiane Etang, Eric Bertrand Fokam, Parfait H. Awono-Ambene

**Affiliations:** 1 Microbiology and Parasitology Department, University of Buea, P.O. Box 63, Buea, Cameroon; 2 Institut de Recherche de Yaoundé, Organisation de Coordination pour la lutte contre les Endémies en Afrique Centrale (OCEAC), B.P. 288 Yaoundé, Cameroon; 3 Centre de Recherches Médicales de Lambaréné (CERMEL), P.O Box 242, Lambaréné, Gabon; 4 Institute of Tropical Medicine, University of Tübingen, Wilhemstrasse 27, P.O. Box 72074, Tübingen, Germany; 5 Laboratory of Animal Biology and Physiology, University of Douala, PO Box 24157, Douala, Cameroon; 6 Laboratory for Biodiversity and Conservation Biology, University of Buea, P.O. Box 63, Buea, Cameroon; 7 Department of Zoology and Animal Physiology, University of Buea, P.O. Box 63, Buea, Cameroon; 8 Department of Biology and Animal Physiology, University of Yaoundé I, P.O. Box 3851, Messa, Yaoundé, Cameroon; 9 Faculty of Medicine and Pharmaceutical Sciences, University of Douala, P.O. Box 2701, Douala, Cameroon

**Keywords:** *Anopheles gambiae* complex, Deltamethrin, Permethrin, Malathion, South-West Region, Cameroon

## Abstract

**Background:**

Members of the *Anopheles gambiae* (*s.l.*) complex are one of the major vectors of malaria in Africa. LLINs and IRS are the most effective tools used in vector control of malaria. However, their effectiveness may be hampered by the development and spread of insecticide resistance in the target vectors species. The objective of this study was to assess the susceptibility of *Anopheles gambiae* (*s.l.*) mosquitoes from South-West Cameroon to deltamethrin, permethrin and to malathion, four years after the mass deployment of LLINs.

**Methods:**

*Anopheles* larvae were collected from Limbe, Tiko and Buea, three cities of the Fako division and reared until adult emergence. Adult mosquitoes from field larvae were identified as belonging to the *Anopheles gambiae* (*s.l.*) complex using standard identification keys. Susceptibility of mosquito samples to deltamethrin, permethrin and malathion was assessed using WHO susceptibility tests protocol for adult mosquitoes. Molecular identification of tested samples was performed using the PCR SINE200 protocol and by PCR-RFLP. The *kdr* alleles were genotyped using the hot ligation oligonucleotide assay (HOLA).

**Results:**

Two species of the *An. gambiae* (*s.l.*) complex, *An. coluzzii* and *An. gambiae* (*s.s.*) were identified in all three study locations with high proportions of *An. coluzzii* in Limbe (84.06%) and Tiko (92.2%), while in Buea, *An. coluzzii* (55.6%) and *An. gambiae* (*s.s.*) (44.4%) occurred almost in the same proportions. Tested samples were found resistant to pyrethroids (deltamethrin and permethrin) in all locations (< 90% mortality), with > 3-fold increase of KDT_50_ values compared with the Kisumu susceptible reference strain of *An. gambiae* (*s.s.*). However, the mosquito populations from Limbe and Buea were fully susceptible to malathion. The L1014F *kdr* was found in both *An. coluzzii* and *An. gambiae* (*s.s.*) with the highest frequencies found in *An. gambiae* (*s.l.*) populations from Tiko (94%) and Buea (90%) compared with the Limbe population (66%) (*P* = 0.00063, *df* = 2). No *kdr* L1014S was observed in analyzed samples.

**Conclusions:**

These findings reemphasize the ongoing development of *An. gambiae* (*s.l.*) resistance to pyrethroids used in impregnating LLINs and suggest the use of malathion as an alternative insecticide for IRS in complementarity with LLINs.

**Electronic supplementary material:**

The online version of this article (10.1186/s13071-018-2979-1) contains supplementary material, which is available to authorized users.

## Background

There are over 500 known species of *Anopheles* among which only 70 are competent vectors of human malaria [1]. Sub-Saharan Africa is the most affected by malaria and this is partly because the ecological conditions of this region are suitable for the most potent vectors of human malaria [2–4], including the *Anopheles funestus* subgroup, i.e. *An. funestus* Giles and the *An. gambiae* (*s.l.*) complex, the latter comprising *An. gambiae* Giles, 1902 [*An. gambiae* (*s.s.*) and *An. coluzzii* [5]] and *An. arabiensis* Patton, 1905 [2, 3]. Three other members of the complex, *An. melas*, *An. merus* and *An. bwambae*, are important vectors in limited geographical areas [6]. Malaria remains the most important parasitic disease in the world, affecting mostly people in developing countries, especially in sub-Saharan Africa. The World Health Organization (WHO) has recently estimated that 840 million people are at risk from malaria, with most of them living in Africa where it is the leading cause of mortality [7]. Furthermore, it is estimated that there were 214 million malaria cases recorded worldwide in 2015 and that the disease led to 438,000 deaths [7]. Sub-Saharan Africa recorded 90% of the malaria mortality, representing a leading cause of deaths among children under 5 years old (70% of all deaths) [7].

Vector control is currently the most effective mass malaria prevention measure. It is centered around two strategies: long-lasting insecticidal nets (LLINs) and indoor residual spraying (IRS) targeting the adults. Five classes of chemical insecticides are recommended for vector control of malaria, namely organochlorides, pyrethroids, organophosphates, carbamates and chlorfenapyr, a pyrolle which recently received an interim approval from the WHO to be used in insecticide-treated nets and in IRS [8, 9]. However, pyrethroids are currently the sole class of insecticides used for net impregnation because of their safety when in contact with human skin and their rapid ‘knockdown’ action on mosquitoes even at low doses, whereas all the four classes of insecticides are recommended for IRS [10].

In Cameroon, mass distribution of LLINs is the main intervention used in vector control of malaria. In addition, since 2004, pregnant women and children under five are entitled to free LLIN distribution through Antenatal Care (ANC) [7]. In 2011, almost 8 million LLINs (PermaNet® 2.0 and OlysetNet®) were distributed in the country [11]. However, the emergence of pyrethroid resistance in *An. gambiae* (*s.l.*) populations from several settings in Cameroon is seen as a threat to ongoing efforts to prevent malaria transmission by LLINs. Furthermore, previous studies identified the mass usage of LLINs as a driving factor in the rise and spread of resistance [12]. Prior to the implementation of LLINs on a large scale in 2011, studies were carried out to determine the susceptibility of mosquitoes in different areas of Cameroon [13–17]. These studies confirmed pyrethroid resistance in *An. gambiae* (*s.l.*) from several locations in Cameroon, both at the phenotypic and the molecular level with the involvement of target site (L1014F *kdr* and L1014S *kdr*) and metabolic resistances. However, few and outdated data [14, 18] are available on the susceptibility of *Anopheles* mosquitoes in the South-West Region of Cameroon, home to the largest agro-industrial complex in the Central African Sub-Region.

Here, we report on the resistance of *An. gambiae* (*s.s.*) and *An. coluzzii* to deltamethrin, permethrin and their susceptibility to malathion in three areas of the South-West Region of Cameroon, four years after a countrywide distribution of LLINs.

## Methods

### Study sites

The study was conducted in Limbe (4°1'30.4"N, 9°11'40.5"E), Tiko (4°4'32.6"N, 9°21'28.9"E) and Buea (4°10'2.4"N, 9°18'27.3"E), three cities of the Fako division in the South-West Region of Cameroon between November and December 2015. The area is subjected to two seasons: a short dry season (November-February) and a long rainy season (March-October) with abundant precipitation [19]. The mean values of the minimum temperatures are 20 °C in December and 18 °C in August; the mean values of the maximum temperatures are 35 °C in August and 30 °C in March [20]. According to the last census, the Region is home to 1,316,079 inhabitants [21].

The area is home to the Cameroon Development Corporation (CDC) which is an agro-industrial company growing crops such as rubber (*Fiscus elastica*) and banana (*Musa* spp.) in Tiko, and oil palm (*Elaeis* spp.) in Tiko and Limbe [22].

The three study areas (Limbe, Tiko and Buea) were chosen because they are among the biggest and most densely populated towns in the South-West Region.

### Mosquitoes sampling

*Anopheles gambiae* (*s.l.*) larvae were collected from developmental sites in each study areas and taken back to the insectary of the Laboratory for Biodiversity and Conservation Biology at the University of Buea, where they were sorted to remove culicines. Anophelines were reared to adult stage and fed with a 10% sugar solution.

### Insecticide susceptibility testing

The susceptibility tests were done according to the WHO standard protocol for adult mosquitoes [12]. Test kits including filter papers impregnated with deltamethrin (0.05%) permethrin (0.75%) and malathion (5%) were purchased from the University Sains Malaysia. Two to three hours before the tests, access of mosquito samples to sugar solution for feeding was interrupted. Two- to five-day-old adult female mosquitoes were morphologically identified using the morphological key of Gillies & de Meillon [2] and Gillies & Coetzee [3] and used for the tests as follows.

Mosquitoes were introduced into observation tubes (20–25 specimens per tube), where they were allowed to rest for an hour. They were then transferred into exposure tubes lined in with insecticide impregnated papers (4 batches of 20–25 mosquitoes per insecticide) for 60 min exposure to insecticides. Specimens knocked down during exposure were recorded at different time intervals (5, 10, 15, 20, 30, 40, 50 and 60 min). Concomitantly, 2 batches of 20–25 mosquitoes were exposed to untreated papers as a control. After exposure to insecticides, the mosquitoes were transferred back into the observation tubes and given access to a 10% sugar solution. The mortality rates were recorded 24 h post-exposure. On completion of the susceptibility test, mosquitoes were transferred into labelled Eppendorf tubes containing silica gel, with control, dead and live mosquitoes kept separately for molecular analyses.

### Molecular identification of mosquitoes

Thirty *An. gambiae* (*s.l.*) per insecticide tested were identified to the species level, i.e. fifteen surviving and fifteen dead An. gambiae (s.l.) were randomly selected for each insecticide. DNA was extracted from single mosquitoes using CTAB 2% using the protocol of Collins et al. [6]. Specimens were identified using the SINE200 PCR protocol of Santolamazza et al. [23] and by PCR-RFLP following the protocol of Fanello et al. [24]. The products were analyzed on a 2% agarose gel.

### *kdr* genotyping

A subsample of 30 *An. coluzzii* and 30 *An. gambiae* (*s.s.*), selected randomly among those killed and those that survived from each town were used to check for the presence of the *kdr* alleles (L1014F and L1014S) using the hot ligation oligonucleotide assay (HOLA) of Lynd et al. [25].

### Data analysis

Data collected were entered in Microsoft Excel 2013. The R software v.3.2.5 [26] was used to compare the mortality rates and the allelic frequencies in the three tested populations using the Chi-square test. We also used the two tailed Fisher’s exact test to compare the chances of mosquitoes surviving the exposure to pyrethroids in the three towns between the species in our study areas. The Windl32 software was used to determine the different knockdown times for 50 and 95% tested samples from each population (KDT_50,_ KDT_95_).

## Results

### Susceptibility of *Anopheles gambiae* (*s.l.*) populations to insecticides

**Fig. 1:**
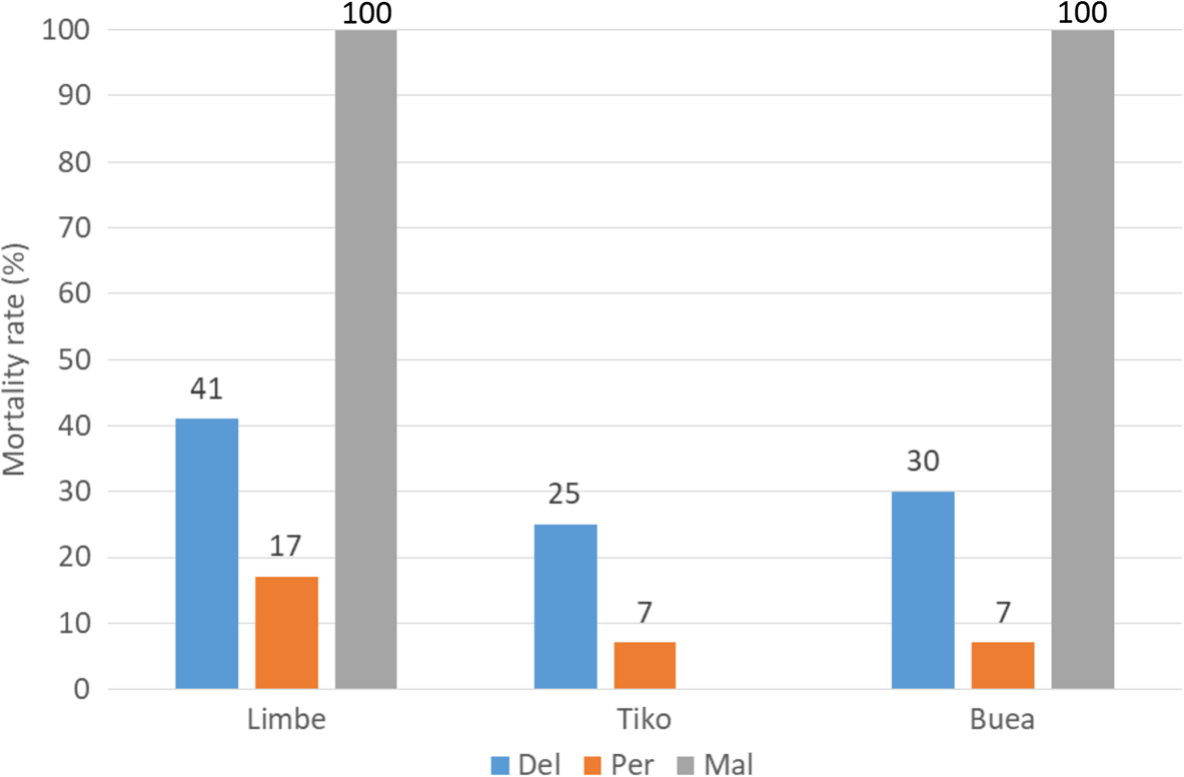
Mortality rates of wild *An. gambiae* (*s.l.*) populations to insecticides tested in the three study sites. *Abbreviations*: Del, deltamethrin (0.5%); Per, permethrin (0.75%); Mal, malathion (5%)

We performed 8 susceptibility tests in total with samples of *Anopheles gambiae* (*s.l.*) collected from Limbe, Buea and Tiko, including 3 tests with deltamethrin, 3 tests with permethrin and 2 tests with malathion. Two to three insecticides were used for each mosquito population. The knockdown times (KDT_50_ and KDT_95_) recorded during exposure to insecticides are summarized in Table 1. The mortality rates recorded 24 h after exposure are given in Fig. 1.

**Table 1:** Times and knockdown ratios of *Anopheles gambiae* (*s.l.*) samples from Limbe, Tiko and Buea to insecticide susceptibility tests

Study site	Insecticides tested	*n*	KDT_50_ (min) (95% CI)	KDT_95_ (min)	Rtkd_50_	Status
Limbe	Deltamethrin (0.05%)	119	29.8 (28.4–31.1)	> 60	3.1	Resistant
	Permethrin (0.75%)	78	> 60	> 60	un	Resistant
Tiko	Deltamethrin (0.05%)	84	46.2 (44.0–48.8)	> 60	4.9	Resistant
	Permethrin (0.75%)	78	> 60	> 60	un	Resistant
Buea	Deltamethrin (0.05%)	147	36.5 (34.9–38.2)	> 60	3.8	Resistant
	Permethrin (0.75%)	88	> 60	> 60	un	Resistant

*Abbreviations*: *n* number tested, *95% CI* 95% confidence interval, *un* undetermined, *KDT*_*50*_ time required for knocking down 50% of individuals, *KDT*_*95*_ time required for knocking down 50% of individuals, *Rtkd*_*50*_ time ratio required for knocking down 50% of individuals

#### Knockdown times

With permethrin, the KDT_50_ were all > 60 min for all the *An. gambiae* (*s.l.*) samples from the three localities. However with deltamethrin, the KDT_50_ were significantly different between the three samples (*P* < 0.05). The highest KDT_50_ was recorded with specimens from Tiko (TKD_50_ > 45 min), followed by those from Buea (TKD_50_ = 36.5 min) and then those from Limbe (TKD_50_ ≈ 30 min). As compared to the KDT_50_ of the Kisumu susceptible strain (9.5 min), the KDT ratios varied from 4.9 to 3.8 and 3.1, respectively, for the Tiko, Buea and Limbe samples. Nevertheless, the KDT_95_ of all the three tested samples to deltamethrin were > 60 min. Conversely, no KDT was recorded in contact with malathion insecticide since organophosphates are not characterized by a knockdown effect.

#### Mortality rates

All the three field mosquito populations were found resistant to deltamethrin and permethrin. The mortality rate to deltamethrin ranged from 25% for the Tiko sample to 41% for the Limbe sample, while to permethrin it was less than 20% in general. The difference in deltamethrin mortality rates between the three samples was significant (*P* = 0.0003, *df* = 2), while no significant difference was seen with permethrin (*P* = 0.1036, *df* = 2).

Tests with malathion were performed only with samples from Limbe and Buea, since there were not enough mosquitoes to perform all the tests in Tiko. Interestingly, the two tested samples were fully susceptible to malathion, with 100% (*n* = 228) mortality rates.

### Mosquito species distribution

Prior to susceptibility tests, all the 822 mosquitoes used for the experiment were morphologically identified as belonging to the *An. gambiae* complex. Then after susceptibility tests, 268 made up of dead and survivors specimens were analyzed by PCR, among which 214 (around 80%) were successfully identified down to species. Two species of the *Anopheles gambiae* complex were recorded: *An. coluzzii* and *An. gambiae* (*s.s.*). *Anopheles coluzzii* was the dominant species in the three towns (*P* = 0.0002594).

*Anopheles coluzzii* was found in an especially great proportion in the Limbe and Tiko samples, representing 84 and 92%, respectively. However, in Buea, although *An. coluzzii* was predominant (55.6%), *An. gambiae* (*s.s.*) were found in greater proportion than in the other two towns (44.4%). There was no statistical difference between *An. coluzzii* and *An. gambiae* (*s.s.*) (Table 2) in terms of their likelihood to survive exposure to pyrethroids in Limbe (*P* = 0.4597) and Tiko (*P* = 1). However, we found that *An. gambiae* (*s.s.*) was less likely to die from exposure to pyrethroids as compared to *An. coluzzi* in Buea (*P* = 0.0073)

**Table 2:** Species distribution of mosquitoes dead or alive after 24 h exposure to deltamethrin and permethrin in Limbe, Tiko and Buea

Species	*An. coluzzii*		*An. gambiae* (*s.s.*)		*P*-value
	No. dead (%)	No. alive (%)	No. dead (%)	No. alive (%)	
Limbe	12 (32.43)	25 (67.57)	5 (50.00)	5 (50.00)	0.4597
Tiko	20 (33.90)	39 (66.10)	1 (20.00)	4 (80.00)	1
Buea	15 (51.72)	14 (48.28)	3 (13.04)	20 (86.96)	0.0073

### *kdr* genotypes

A total of 134 specimens were genotyped for *kdr* L1014F and L1014S mutations as the number of *An. gambiae* (*s.s.*) did not reach 30 in Limbe and Tiko. Those genotyped included 87 *An. coluzzii* and 47 *An. gambiae* (*s.s.*) randomly selected from the three study areas (Table 3).

**Table 3:** Distribution of the *kdr* genotypes within *An. coluzzii* and *An. gambiae* (*s.s.*) from Limbe, Tiko and Buea

Study sites	Species	*n*	L1014F *kdr* mutation			F_1_ (L1014F) (%)	F_2_ (L1014L) (%)	F_1_^’^ (L1014F) (%)	F_2_^’^ (L1014L) (%)
			SS	SR	RR				
Limbe	*An. coluzzii*	30	2	14	14	70	30	66^a^	34
	*An. gambiae* (*s.s.*)	10	1	7	2	55	45		
Tiko	*An. coluzzii*	27	1	2	24	93	7	94^b^	6
	*An. gambiae* (*s.s.*)	5	0	0	5	100			
Buea	*An. coluzzii*	30	1	7	22	85	15	90^b^	10
	*An. gambiae* (*s.s.*)	32	0	4	28	94	6		

*Note*: Values with different superscript letters are significantly different

*Abbreviations*: *F*_*1*_ and *F*_*2*_ allelic frequencies of L1014F *kdr* in *An. coluzzii* and *An. gambiae *(*s.s*.), *F*_*1*_ and*’ F*_*2*_*’* allelic frequencies of L1014F *kdr* in *An. gambiae* (*s.l*.)

The L1014F *kdr* was present in both *An. coluzzii* and *An. gambiae* (*s.s.*) from the three locations. Its allelic frequencies were high (> 80%) both for *An. coluzzii* and *An. gambiae* (*s.s.*) (*χ*^2^ = 0.186, *P* = 0.67, *df* = 1), except in *An. gambiae* (*s.s.*) from Limbe which displayed a lower frequency (55%). However, the *kdr* L1014F allelic frequencies in *An. gambiae* (*s.l.*) populations were significantly higher in those from Tiko and Buea (90–94%) compared with the mosquito population from Limbe (66%) (*χ*^2^= 14.73, *P* = 0.00063, *df* = 2). No *kdr* L1014S was seen in analyzed samples. Although, the frequency of the *kdr* L1014F was higher in resistant mosquitoes (85.38%) than in susceptible mosquitoes (80.26%), the difference was not statistically significant (*P* = 0.5622).

## Discussion

Current malaria vector control in a number of countries mainly relies on the use of LLINs due to their cost-effectiveness [9]. Malaria vector resistance to pyrethroid insecticides that are used for net impregnation has risen and is spreading, especially across Africa [27–31], including Cameroon [13–18].

Previous studies conducted in Tiko revealed a change in insecticide susceptibility of the local *An. gambiae* (*s.l.*) population from a fully susceptible status to deltamethrin and permethrin [14] to reduced susceptibility to deltamethrin, but still full susceptibility to permethrin [18]. Results from the current study clearly indicate an increase in deltamethrin and permethrin resistance in this vector population with mortality lower than 30%. The four- to five-fold increase in KDT_50_ when compared to the susceptible Kisumu reference strain of *An. gambiae* (*s.s.*) and to the KDT_50_ (≈ 12 min) recorded by Ndjemaï et al. [18] suggests the implication of target site as a possible mechanism responsible for the resistance to pyrethroids. In fact, high KDT_50_ in field mosquito populations has been suggested to provide a sensitive indicator of the implication of *kdr* mutations in phenotypic resistance to pyrethroids [12, 32].

Similar evolvement of pyrethroid resistance is applicable to *An. gambiae* (*s.l.*) populations from Limbe and Buea. In those two localities, *An. gambiae* (*s.l.*) were found to be fully susceptible to deltamethrin and permethrin in 2007 [14] and are now resistant to pyrethroids. Although the wide use of LLINs is known to exert selective pressure on local malaria vector populations, the use of insecticides for other purposes such as agriculture could also play a role in the increase of pyrethroid resistance in the three study areas. The South-West Region shelters intensive agriculture, with many plantation areas belonging to the Cameroon Development Corporation (CDC). The role of agricultural use of insecticides in plantations on the selection of *An. gambiae* resistance to insecticides has previously been reported in Burkina Faso [28] and Cameroon [33]. On the other hand, studies conducted in Benin [34] and Burkina Faso [35] showed that three years after the distribution of LLINs, there was an increase in the frequencies of the *kdr* mutation in local *An. gambiae* (*s.l.*) populations associated with deltamethrin resistance. Thus, the mass distribution of LLINs impregnated with deltamethrin (PermaNet® 2.0) and permethrin (OlysetNet®) throughout Cameroon in 2011, could have played a role in the development of resistance of *Anopheles* populations from these three towns.

Although five classes of insecticides are used for malaria vector control, only pyrethroids are currently used in impregnating bed nets. The pyrolle chlorfenapyr recently received an interim approval for it to be used in bed nets. This insecticide is the first to receive an interim approval from the WHO in more than 30 years and has been shown to be effective against pyrethroid resistant mosquitoes [9]. The remaining three (carbamates, organophosphates and organochlorides) are used for IRS. However, despite the fact that chlorfenapyr increases the list of insecticides available for malaria vector control, these insecticides are still few. Therefore, there is the need to develop strategies aimed at keeping the pool of currently available insecticides as effective as possible. The simultaneous use of two insecticides (combination strategy) has been recommended as a resistance management strategy which aims at killing resistant vectors [8]. The assumption is that although mosquitoes may be resistant to one insecticide they may still be killed by another one from the same class or form another class [8]. The simultaneous use of pyrethroids in LLINs and net wall hanging (NWH) treated with organophosphate (p-methyl) was shown to improve malaria control by increasing blood feeding inhibition and personal protection in areas with pyrethroids-resistant mosquitoes in Burkina Faso [36] and Tanzania [37]. However, in areas with multiple resistance in Côte d’Ivoire, combination of the insecticides cited above failed to improve malaria control [38]. In Limbe and Buea where mosquitoes were tested for both classes of insecticides, full susceptibility to malathion was demonstrated and this insecticide could be used for IRS in addition to LLINs. Therefore, the combination of LLINs and IRS with malathion (or treated NWH) could be trialed in South-West Cameroon as a resistance management strategy. The implementation of measures to supplement the distribution of LLINs is of more concern as it was shown that most of the LLINs distributed during the first national distribution campaign had lost both their physical integrity and insecticidal efficacy especially in Limbe and Buea [39].

All the mosquitoes identified morphologically were found to be *An. gambiae* (*s.l.*) which is in conformity with previous findings in the areas [14, 18]. Molecular analysis of tested samples revealed two species of the *An. gambiae* complex, *An. coluzzii* and *An. gambiae* (*s.s.*) Their proportions in the three study sites were in accordance with previous reports [40] with a greater proportion of *An. coluzzii* found in the urbanized towns of Tiko and Limbe as compared to Buea where mosquito samples were collected in a rural setting. Even though a considerable proportion of mosquitoes encountered in Buea were still *An. coluzzii*, the proportion of *An. gambiae* (*s.s.*) was greater than in the two other towns. These two species have previously been shown to be distributed along an urbanization gradient [40] with more *An. coluzzii* usually found in urbanized areas as compared to *An. gambiae* (*s.s.*) which are usually prevalent in rural areas [40]. The low proportion of *An. gambiae* (*s.s.*) in Tiko and Limbe could be explained by the fact that larvae were collected at the end of the rainy season, as this species prefers small, temporal and clean developmental sites formed by the accumulation of rain water whereas *An. coluzzii* is usually found in permanent developmental sites such as those resulting from human activities in urban areas [41]. However, although Limbe and Tiko are close to the Atlantic coast, no *An. melas* was identified in analyzed mosquito samples as further confirmed by the PCR-PFLP protocol of Fanello et al. [24] applied to the samples found to be *An. gambiae* (*s.s.*) by the SINE 200 PCR protocol [23] from these two towns. During larval collections, *An. gambiae* (*s.l.*) was found in the same breeding sites with *Culex* mosquitoes as reported by Kamdem et al. [40], stemming from the fact that *An. coluzzii* has adapted to highly polluted larval habitats. This distribution of species may have epidemiological consequences such that in urban settings there will be a year-round malaria transmission mediated by *An. coluzzii*, while in rural settings transmission it may be more seasonal [42]. The failure to amplify some individuals could be a consequence of poor DNA extraction and possibly DNA degradation linked to the storage of the samples as previously reported [43]. Although, there was no statistical difference in the likelihood of *An. coluzzii* and *An. gambiae* (*s.s.*) surviving exposure to pyrethroids in Limbe and Tiko, *An. gambiae* (*s.s.*) was more likely to survive such exposure as compared to *An. coluzzii* in Buea*.* Similar results were obtained by Badolo et al. [35] in Burkina Faso where they also found that *An. gambiae* (*s.s.*) had the best chance of surviving DDT or permethrin exposure as compared to *An. coluzzii.* This result emphasizes the need to monitor how species distribution and change over the years may influence resistance of mosquitoes to pyrethroids.

*Kdr* mutations in mosquito populations from Cameroon was first reported by Etang et al. [13]. Since then many studies have reported this mutation in different areas of the country [15–17, 33, 44]. The L1014F *kdr* mutations were present in *An. coluzzii* and *An. gambiae* (*s.s.*) populations from the three study sites. However, no *kdr* L1014S was seen in analyzed samples which are similar in Tiko to results from a previous report [45]. The high frequencies of L1014F *kdr* mutation in screened individuals in Tiko and Buea (90–94%) suggests that the allele is almost fixed following an increase from around 3% in Tiko as reported by Nwane et al. [45] in 2011. However, in Limbe where mosquitoes were more susceptible to deltamethrin, the *kdr* 1014F frequency (66%) was significantly lower than those recorded in Tiko and Buea. These results, especially in Tiko where the four- to five-fold increase in KDT_50_ was accompanied by an increase in *kdr* frequency from 3 to 90%, suggest the involvement of the *kdr* mutation in the phenotypic resistance. Although the frequency of the *kdr* mutation in resistant mosquitoes to pyrethroids was higher than in susceptible mosquitoes, this was not statistically significant. This may be due to the small number of samples screened for this mutation (Additional file 1: Table S1). However, the involvement of additional resistance mechanism in the phenotypic resistance such as P450s or esterases cannot be overruled as previously reported [34, 35].

## Conclusions

The present study revealed an increase in pyrethroid resistance and high levels of *kdr* L1014F allelic frequencies in *An. coluzzii* and *An. gambiae* (*s.s.*) from Limbe, Tiko and Buea in the South-West Region of Cameroon. However, the involvement of other resistance mechanisms cannot be overruled and need to be further investigated as well as the impact of large scale LLIN campaigns on the development of insecticide resistance. The fact that mosquitoes were fully susceptible to malathion in Limbe and Buea gives the possibility for this insecticide to be used for IRS in combination with LLINs, as a potential resistance management strategy for the study area.

## Additional file

Additional file 1: **Table S1.** Molecular identification and *kdr* genotypes of samples from Limbe, Tiko and Buea. (PDF 84 kb)

## Abbreviations

BUCREP: Bureau Central des Recensements et des Etudes de Population
CDC: Cameroon Development Corporation
DDT: Dichlorodiphenyltrichloroethane
DNA: Deoxyribonucleic acid
HOLA: Hot ligation oligonucleotide assay
IRS: Indoor residual spraying
*kdr*: Knockdown resistance gene
KdT: Knockdown time
LLIN: Long-lasting insecticidal net
NWH: Net wall hanging
OCEAC: Organisation de Coordination pour la lutte contre les Endémies en Afrique Centrale
PCR: Polymerase chain reaction
WHO: World Health Organization
