# Supplementary material for: Insecticide susceptibility status of Anopheles gambiae (s.l.) in South-West Cameroon four years after long-lasting insecticidal net mass distribution
Source: Parasit Vectors. 2018 Jul 4;11:391. doi: 10.1186/s13071-018-2979-1 (PMC6033221; doi:10.1186/s13071-018-2979-1)
Supplement: Supplementary file 1 — Table S1. Molecular identification and kdr genotypes of samples from Limbe, Tiko and Buea. (PDF 84 kb) [file 13071_2018_2979_MOESM1_ESM.pdf]

Table S1: Molecular identification and Kdr genotypes of samples from Limbe, Tiko and Buea

Study site	Insecticide	code	Status	Molecular identification	Kdr genotype
Muea	delta	Mu_2	dead	*An. coluzzii*	wFwF
Muea	delta	Mu_3	dead	*An. coluzzii*	wFwF
Muea	delta	Mu_4	dead	*An. coluzzii*	wFwF
Muea	delta	Mu_5	dead	*An. coluzzii*	wFwF
Muea	delta	Mu_6	dead	*An. coluzzii*	wLwF
Muea	delta	Mu_7	dead	*An. coluzzii*	wFwF
Muea	delta	Mu_8	dead	*An. coluzzii*	wFwF
Muea	delta	Mu_9	dead	*An. coluzzii*	wFwF
Muea	delta	Mu_10	dead	*An. coluzzii*	wLwF
Muea	delta	Mu_11	dead	*An. gambiae (s.s.)*	wFwF
Muea	delta	Mu_12	dead	*An. coluzzii*	wLwF
Muea	delta	Mu_14	dead	*An. coluzzii*	wFwF
Muea	delta	Mu_29	alive	*An. coluzzii*	wFwF
Muea	delta	Mu_30	alive	*An. gambiae (s.s.)*	wFwF
Muea	delta	Mu_31	alive	*An. gambiae (s.s.)*	wFwF
Muea	delta	Mu_32	alive	*An. gambiae (s.s.)*	wFwF
Muea	delta	Mu_33	alive	*An. gambiae (s.s.)*	wFwF
Muea	delta	Mu_34	alive	*An. gambiae (s.s.)*	wFwF
Muea	delta	Mu_35	alive	*An. gambiae (s.s.)*	wFwF
Muea	delta	Mu_36	alive	*An. gambiae (s.s.)*	wFwF
Muea	delta	Mu_37	alive	*An. gambiae (s.s.)*	wFwF
Muea	delta	Mu_38	alive	*An. gambiae (s.s.)*	wFwF
Muea	delta	Mu_83	alive	*An. coluzzii*	wFwF
Muea	delta	Mu_84	alive	*An. gambiae (s.s.)*	wFwF
Muea	delta	Mu_85	alive	*An. gambiae (s.s.)*	wFwF
Muea	perm	Mu_144	dead	*An. coluzzii*	wFwF
Muea	perm	Mu_145	dead	*An. gambiae (s.s.)*	wFwF
Muea	perm	Mu_146	dead	*An. coluzzii*	wFwF
Muea	perm	Mu_147	dead	*An. gambiae (s.s.)*	wFwF
Muea	perm	Mu_152	alive	*An. gambiae (s.s.)*	wFwF
Muea	perm	Mu_153	alive	*An. coluzzii*	wFwF
Muea	perm	Mu_155	alive	*An. gambiae (s.s.)*	wFwF
Muea	perm	Mu_157	alive	*An. gambiae (s.s.)*	wFwF
Muea	perm	Mu_167	alive	*An. gambiae (s.s.)*	wLwF
Muea	perm	Mu_173	alive	*An. gambiae (s.s.)*	wFwF
Muea	perm	Mu_174	alive	*An. coluzzii*	wLwL
Muea	perm	Mu_182	alive	*An. coluzzii*	wFwF
Muea	perm	Mu_183	alive	*An. coluzzii*	wLwF
Muea	perm	Mu_186	alive	*An. coluzzii*	wLwF
Muea	perm	Mu_187	alive	*An. coluzzii*	wFwF
Muea	perm	Mu_189	alive	*An. coluzzii*	wFwF
Muea	perm	Mu_192	alive	*An. gambiae (s.s.)*	wFwF
Muea	malathion	Mu_219	dead	*An. gambiae (s.s.)*	wLwF

Muea	malathion	Mu_223	dead	*An. coluzzii*	wLwF
Muea	malathion	Mu_225	dead	*An. coluzzii*	wFwF
Muea	malathion	Mu_227	dead	*An. gambiae (s.s.)*	wLwF
Muea	malathion	Mu_229	dead	*An. gambiae (s.s.)*	wFwF
Muea	malathion	Mu_233	dead	*An. gambiae (s.s.)*	wLwF
Muea	malathion	Mu_235	dead	*An. gambiae (s.s.)*	wFwF
Muea	malathion	Mu_236	dead	*An. gambiae (s.s.)*	wFwF
Muea	malathion	Mu_237	dead	*An. coluzzii*	wFwF
Muea	malathion	Mu_239	dead	*An. gambiae (s.s.)*	wFwF
Muea	malathion	Mu_240	dead	*An. gambiae (s.s.)*	wFwF
Muea	malathion	Mu_241	dead	*An. coluzzii*	wFwF
Muea	malathion	Mu_242	dead	*An. gambiae (s.s.)*	wFwF
Muea	malathion	Mu_243	dead	*An. gambiae (s.s.)*	wFwF
Muea	malathion	Mu_244	dead	*An. coluzzii*	wFwF
Muea	malathion	Mu_246	dead	*An. gambiae (s.s.)*	wFwF
Muea	malathion	Mu_248	dead	*An. coluzzii*	wFwF
Muea	malathion	Mu_249	dead	*An. coluzzii*	wLwF
Muea	malathion	Mu_250	dead	*An. coluzzii*	wFwF
Muea	perm	Mu_262	alive	*An. gambiae (s.s.)*	wFwF
Tiko	delta	Ti_1	alive	*An. gambiae (s.s.)*	wFwF
Tiko	delta	Ti_2	alive	*An. coluzzii*	wFwF
Tiko	delta	Ti_6	alive	*An. coluzzii*	wFwF
Tiko	delta	Ti_8	alive	*An. coluzzii*	wFwF
Tiko	delta	Ti_9	alive	*An. coluzzii*	wFwF
Tiko	delta	Ti_12	dead	*An. coluzzii*	wFwF
Tiko	delta	Ti_15	dead	*An. coluzzii*	wFwF
Tiko	delta	Ti_17	dead	*An. coluzzii*	wFwF
Tiko	delta	Ti_19	dead	*An. coluzzii*	wFwF
Tiko	delta	Ti_21	dead	*An. coluzzii*	wFwF
Tiko	delta	Ti_23	dead	*An. gambiae (s.s.)*	wFwF
Tiko	delta	Ti_24	dead	*An. coluzzii*	wFwF
Tiko	delta	Ti_26	dead	*An. coluzzii*	wFwF
Tiko	delta	Ti_30	alive	*An. coluzzii*	wFwF
Tiko	delta	Ti_32	alive	*An. coluzzii*	wLwF
Tiko	delta	Ti_39	alive	*An. coluzzii*	wLwL
Tiko	delta	Ti_54	alive	*An. gambiae (s.s.)*	wFwF
Tiko	delta	Ti_55	alive	*An. coluzzii*	wFwF
Tiko	perm	Ti_81	alive	*An. coluzzii*	wFwF
Tiko	perm	Ti_89	alive	*An. coluzzii*	wFwF
Tiko	perm	Ti_92	alive	*An. coluzzii*	wFwF
Tiko	perm	Ti_95	alive	*An. gambiae (s.s.)*	wFwF
Tiko	perm	Ti_101	alive	*An. coluzzii*	wFwF
Tiko	perm	Ti_108	alive	*An. coluzzii*	wFwF
Tiko	perm	Ti_114	alive	*An. coluzzii*	wLwF
Tiko	perm	Ti_121	alive	*An. coluzzii*	wFwF
Tiko	perm	Ti_125	alive	*An. gambiae (s.s.)*	wFwF

Tiko	perm	Ti_127	alive	*An. coluzzii*	wFwF
Tiko	perm	Ti_135	alive	*An. coluzzii*	wFwF
Tiko	perm	Ti_144	dead	*An. coluzzii*	wFwF
Tiko	perm	Ti_146	dead	*An. coluzzii*	wFwF
Tiko	perm	Ti_148	dead	*An. coluzzii*	wFwF
Limbé	delta	Li_6	alive	*An. coluzzii*	wFwF
Limbé	delta	Li_10	dead	*An. coluzzii*	wLwF
Limbé	delta	Li_11	dead	*An. coluzzii*	wLwL
Limbé	delta	Li_16	dead	*An. coluzzii*	wLwF
Limbé	delta	Li_17	dead	*An. coluzzii*	wLwF
Limbé	delta	Li_20	dead	*An. coluzzii*	wLwF
Limbé	delta	Li_21	dead	*An. coluzzii*	wLwF
Limbé	delta	Li_32	alive	*An. coluzzii*	wLwF
Limbé	delta	Li_35	alive	*An. coluzzii*	wFwF
Limbé	delta	Li_36	alive	*An. coluzzii*	wFwF
Limbé	delta	Li_42	alive	*An. coluzzii*	wFwF
Limbé	delta	Li_82	alive	*An. coluzzii*	wFwF
Limbé	delta	Li_85	alive	*An. coluzzii*	wLwF
Limbé	delta	Li_92	alive	*An. coluzzii*	wLwL
Limbé	delta	Li_93	alive	*An. gambiae (s.s.)*	wFwF
Limbé	delta	Li_96	alive	*An. gambiae (s.s.)*	wLwF
Limbé	delta	Li_97	dead	*An. gambiae (s.s.)*	wLwF
Limbé	delta	Li_102	dead	*An. gambiae (s.s.)*	wLwF
Limbé	perm	Li_115	dead	*An. coluzzii*	wFwF
Limbé	perm	Li_118	alive	*An. coluzzii*	wLwF
Limbé	perm	Li_121	alive	*An. gambiae (s.s.)*	wFwF
Limbé	perm	Li_122	alive	*An. coluzzii*	wLwF
Limbé	perm	Li_142	alive	*An. gambiae (s.s.)*	wLwF
Limbé	perm	Li_143	alive	*An. coluzzii*	wLwF
Limbé	perm	Li_147	alive	*An. gambiae (s.s.)*	wLwF
Limbé	perm	Li_148	alive	*An. coluzzii*	wFwF
Limbé	perm	Li_181	dead	*An. gambiae (s.s.)*	wLwL
Limbé	perm	Li_191	dead	*An. gambiae (s.s.)*	wLwF
Limbé	malathion	Li_219	dead	*An. coluzzii*	wLwF
Limbé	malathion	Li_226	dead	*An. coluzzii*	wFwF
Limbé	malathion	Li_230	dead	*An. coluzzii*	wFwF
Limbé	malathion	Li_237	dead	*An. coluzzii*	wLwF
Limbé	malathion	Li_245	dead	*An. coluzzii*	wLwF
Limbé	malathion	Li_265	dead	*An. coluzzii*	wFwF
Limbé	malathion	Li_271	dead	*An. coluzzii*	wFwF
Limbé	malathion	Li_285	dead	*An. coluzzii*	wFwF
Limbé	malathion	Li_296	dead	*An. gambiae (s.s.)*	wLwF
Limbé	malathion	Li_297	dead	*An. coluzzii*	wLwF
Limbé	malathion	Li_307	dead	*An. coluzzii*	wFwF
Limbé	malathion	Li_316	dead	*An. coluzzii*	wFwF
